# Smoothened Adopts Multiple Active and Inactive Conformations Capable of Trafficking to the Primary Cilium

**DOI:** 10.1371/journal.pone.0005182

**Published:** 2009-04-13

**Authors:** Christopher W. Wilson, Miao-Hsueh Chen, Pao-Tien Chuang

**Affiliations:** Cardiovascular Research Institute, University of California San Francisco, San Francisco, California, United States of America; Universidade Federal do Rio de Janeiro (UFRJ), Instituto de Biofísica da UFRJ, Brazil

## Abstract

Activation of Hedgehog (Hh) signaling requires the transmembrane protein Smoothened (Smo), a member of the G-protein coupled receptor superfamily. In mammals, Smo translocates to the primary cilium upon binding of Hh ligands to their receptor, Patched (Ptch1), but it is unclear if ciliary trafficking of Smo is sufficient for pathway activation. Here, we demonstrate that cyclopamine and jervine, two structurally related inhibitors of Smo, force ciliary translocation of Smo. Treatment with SANT-1, an unrelated Smo antagonist, abrogates cyclopamine- and jervine-mediated Smo translocation. Further, activation of protein kinase A, either directly or through activation of Gαs, causes Smo to translocate to a proximal region of the primary cilium. We propose that Smo adopts multiple inactive and active conformations, which influence its localization and trafficking on the primary cilium.

## Introduction

The Hedgehog (Hh) signal transduction cascade is critical for many aspects of embryonic development, and aberrant regulation of the pathway results in a wide variety of congenital defects and cancers [1]. Activation of the pathway is initiated by binding of Hh ligand to the core receptor Patched (Ptch1 in mammals), a twelve-pass membrane protein with distant similarity to the resistance-nodulation division (RND) of bacterial transporters [2]. The interaction of Hh with Ptch1 relieves inhibition of Smoothened (Smo), a seven-pass transmembrane protein with structural similarity to G-protein coupled receptors (GPCRs), via unknown mechanisms. Once released from Ptch1-mediated inhibition, Smo communicates the status of pathway activation to the Ci/Gli transcription factors, which commence transcription of Hh target genes. This is achieved through the production of Gli activators, derived from full-length Gli proteins, and a concomitant reduction in levels of Gli repressors resulting from limited proteolysis of full-length Gli proteins [3]. The mechanistic details of Smo activation are unclear and may differ between invertebrates and vertebrates [4], [5]. In addition, the means by which Smo relays the status of pathway activation to the Gli proteins do not appear to be evolutionarily conserved [4], particularly the cellular microenvironment in which Smo is activated and the downstream components it interacts with. Nevertheless, two general features of Smo activation that are shared between species are a change in its subcellular distribution after relief of Ptch1 inhibition [6], [7], and conformational changes in the extracellular and cytosolic domains [8]. A conserved series of arginine (Arg) residues in the C-tail of both fly and mammalian Smo plays a critical role in modulation of conformation. How these events lead to Smo activation remains a central unresolved issue in understanding the molecular mechanisms of Hh signaling.

In mammals, the primary cilium is essential for proper interpretation of the Hh signal. Cilia contain a long microtubular axoneme, extending from the basal body and surrounded by an external membrane that is continuous with the plasma membrane. Assembly and maintenance of the primary cilium are mediated by the process called intraflagellar transport (IFT), which involves bidirectional movement of IFT particles powered by anterograde kinesin (Kif3a, b and c) and retrograde dynein motors [9], [10]. Mutations that abolish the biogenesis or function of the primary cilium lead to defective Hh signaling [11]. Further, the production of both Gli activators and repressors is affected in the absence of the cilium, leading to a loss of Gli repressive activity without a corresponding gain of transcriptional activation [10], [12], [13]. Smo localization to the primary cilium is associated with Hh pathway activation, and other components of the pathway, including Gli proteins and Ptch1, are also found in this organelle [14], [15]. Mutations in Smo that confer constitutive Hh pathway activation (SmoA1) promote ciliary localization of Smo in the absence of Hh stimulation; conversely mutations that abolish ciliary localization (CLDSmo) appear to render the protein incapable of activating the pathway in the presence of the primary cilium [7]. Ptch1 localizes to the cilium in the absence of Hh ligand, and traffics off the cilium after Hh binding, allowing movement of Smo to the axoneme [15]. It has been proposed that the cilium acts as a scaffold or provides a specialized microenvironment for relaying the Hh signal [10], [16]. This led to a model in which Smo adopts an active conformation upon localizing to the primary cilium, which is capable of coupling to yet-to-be identified downstream components, thus resulting in stimulation of Gli activators, reduction in Gli repressors, and induction of target gene expression. Here, we show that a distinct class of Smo antagonists which suppress Smo-mediated pathway activation also unexpectedly stimulate translocation of Smo to the primary cilium. In addition, modulation of protein kinase A (PKA) activity by chemical means causes a partial accumulation of Smo on a proximal segment of the primary cilium. We propose that multiple conformational changes of Smo are required for ciliary translocation and subsequent pathway activation.

## Results and Discussion

### Smo localizes to the cilium upon both activation and repression of the Hh pathway

**Figure 1:**
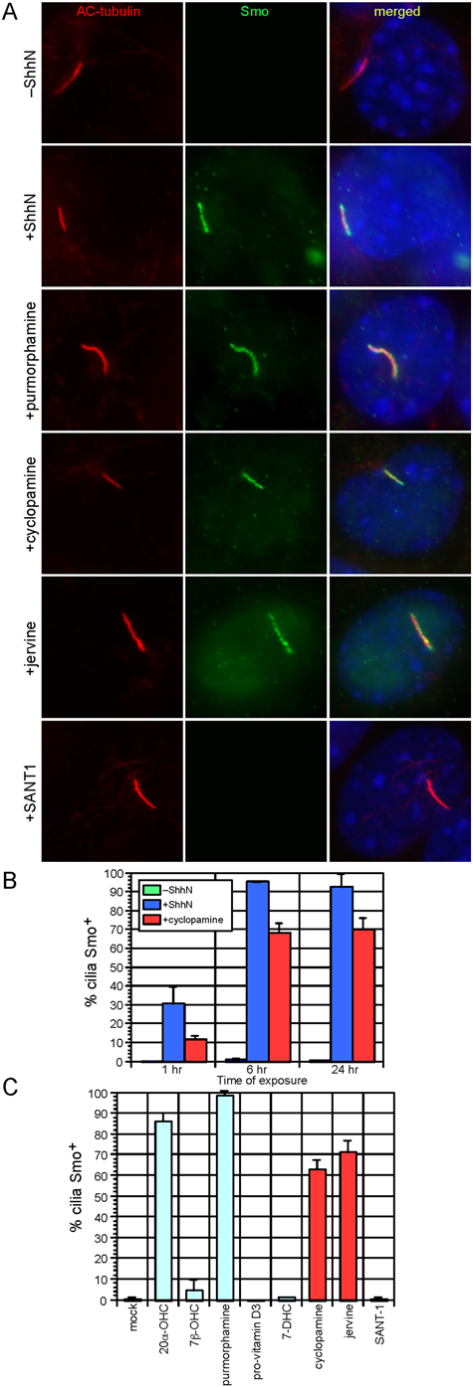
Hh pathway agonists and antagonists stimulate Smo translocation to the primary cilium. (A) Wild-type mouse embryonic fibroblasts (MEFs) stained with antibodies against endogenous Smo (green), acetylated (AC) tubulin (red), and DAPI (blue). MEFs treated with ShhN-conditioned media (ShhN-CM), purmorphamine, cyclopamine, and jervine display Smo staining along the entire length of the cilium. Note that fixation with paraformaldehyde produces artifactual nuclear staining with the Smo antibody; this nuclear staining is not visible after fixation in methanol (See Fig. 3C). (B) Quantification of the percentage of Smo-positive (Smo^+^) cilia in MEFs after treatment with ShhN CM and cyclopamine for the indicated times. Cyclopamine induces Smo translocation to a significant number of cilia after 6 hours of treatment, although this is less than that induced by ShhN CM. All quantification of cilia staining in this and subsequent figures represents at least two separate experiments with a minimum of 100 cilia scored per time point and condition. Error bars indicate +/− standard deviation (SD). (C) Quantification of Smo^+^ cilia in MEFs after treatment with indicated compounds for 24 hours. 20-α-hydroxysterol (20α-OHC), purmorphamine, cyclopamine, and jervine induce Smo translocation to the cilium. Error bars indicate +/− SD.

We generated antibodies against the C-terminal domain of mouse Smo [17] to examine the ciliary localization of endogenous Smo in response to known Hh pathway agonists and antagonists. When exposed to conditioned media (CM) collected from cells expressing the N-terminal signaling fragment of Sonic hedgehog (ShhN), wild-type mouse embryonic fibroblasts (MEFs) accumulated Smo in primary cilia, and nearly 100% of cilia were positive for Smo (Smo^+^) after 6 hours of treatment (Fig. 1A, 1B). In agreement with previously published results [7], a reduced number of cilia were Smo^+^ after brief (1 hour) treatment with cyclopamine, a teratogen derived from the *Veratrum* genus of plants and a well-defined Smo antagonist known to bind to the heptahelical bundle of Smo (Fig. 1A, 1B) [18]. Surprisingly, prolonged treatment of MEFs with cyclopamine resulted in a significant number of Smo^+^ cilia, with roughly 70% displaying strong Smo signal along the entire length of the cilium after 24 hours of exposure to cyclopamine (Fig. 1A, 1B). We speculate that the increased time of cyclopamine treatment required to generate a high number of Smo^+^ cilia underlies the difference between our observation and prior reports. This finding also provided a unique opportunity to examine the relationship between ciliary localization of Smo and Hh pathway activation.

**Figure 2 pone-0005182-g002:**
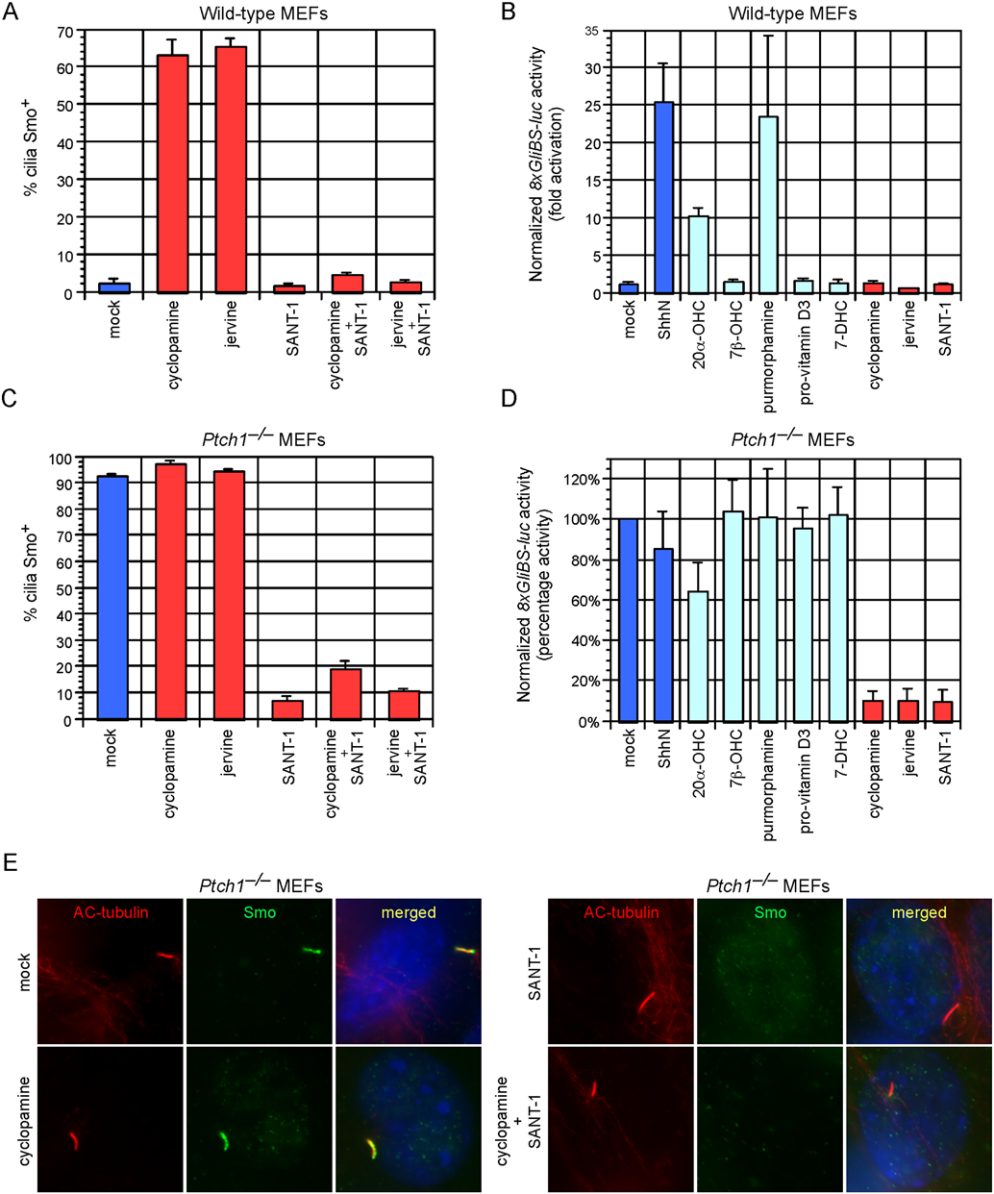
SANT-1 inhibits cyclopamine-and jervine- induced Smo translocation to the primary cilium. (A) Quantification of Smo^+^ cilia in wild-type MEFs after treatment with indicated compounds for 24 hours. SANT-1 inhibits cyclopamine- and jervine-induced ciliary translocation of Smo. Error bars indicate +/− SD. (B) Fold activation of *8xGliBS-luciferase* Hh reporter in wild-type MEFs after agonist and antagonist treatment for 48 hours. Treatment with ShhN-CM, 20-α-hydroxysterol (20α-OHC), or purmorphamine [but not 7β-hydroxysterol (7β-OHC), pro-vitamin D3, 7-dehydrocholesterol (7-DHC), cyclopamine, jervine or SANT-1] activated a Gli transcriptional response. Results are representative of three experiments in two wild-type MEF lines, and were normalized to a constitutively active *Renilla* luciferase reporter. Error bars indicate +/− SD. (C) Quantification of Smo^+^ cilia in *Ptch1* ^−/−^ MEFs after treatment for 24 hours. Cyclopamine and jervine do not disrupt constitutive Smo localization. SANT-1 removes Smo from the cilium of *Ptch1* ^−/−^ MEFs in the presence or absence of cyclopamine and jervine. Error bars indicate +/− SD. (D) Percentage activity of *8xGliBS-luciferase* reporter in *Ptch1* ^−/−^ MEFs after treatment for 48 hours. Cyclopamine, jervine or SANT-1 efficiently block Hh responses. Note that 7-DHC and pro-vitamin D3 do not significantly inhibit the activity of the reporter. Results are the mean of three independent experiments, and were normalized to a constitutively active *Renilla* luciferase reporter. Error bars indicate +/− SD. (E) *Ptch1* ^−/−^ MEFs stained with antibodies against acetylated (AC) tubulin (red), Smo (green) and DAPI (blue). SANT-1 removes Smo from the cilium of *Ptch1* ^−/−^ MEFs in the presence or absence of cyclopamine.

This unexpected result prompted us to screen a number of previously reported modulators of Smo activity for their ability to induce or prevent Smo translocation to the primary cilium. The Smo agonists 20-α-hydroxysterol [19] and purmorphamine [20] caused a significant translocation of Smo to the cilium, comparable to that seen after treatment with ShhN-CM (Fig. 1A, 1C) [15]. As previously described, the related oxysterol, 7β-hydroxysterol, neither induced Smo translocation nor activated the pathway (Fig. 1C, Fig. 2B) [15]. 7-dehydrocholesterol and its metabolite, pro-vitamin D3, had no effect on the subcellular distribution of Smo (Fig. 1C) although pro-vitamin D3 was proposed to be transported by Ptch1 to inhibit Gli activity [21], [22]. Notably, jervine, a close chemical relative of cyclopamine, was also able to induce trafficking of Smo to the primary cilium (Fig. 1A, 1C). By contrast, SANT-1, a structurally unrelated Smo antagonist [23], did not induce Smo trafficking to the cilium (Fig 1A, 1C). We next correlated induction of Smo ciliary translocation with activation of endogenous Gli transcription factors. Measurement of activation of a firefly luciferase reporter driven by eight multimerized Gli-binding sites (8xGliBS-luc) [24] in wild-type MEFs showed that only treatment with ShhN-CM, 20-α-hydroxysterol, or purmorphamine (but not cyclopamine, jervine, SANT1 or pro-vitamin D3) activated a Gli transcriptional response (Fig. 2B). Taken together, the data indicate that while ciliary translocation of Smo can be associated with Gli transcriptional activation, trafficking to the axoneme is not sufficient for Hh pathway activation. Moreover, as cyclopamine is not known to affect the subcellular distribution of Ptch1, our results suggest that Ptch1 may co-exist with inactive Smo conformations on the ciliary axoneme. Recent studies of Ptch1 and Smo trafficking have revealed that Smo bound to a Hh agonist, SAG, is found on the cilium with Ptch1 [15]. We hypothesize that both inactive and active states of Smo may be decoupled from Ptch1-mediated inhibition of ciliary trafficking and Smo activation on the cilium thus requires additional steps.

### SANT-1 inhibits cyclopamine- and jervine-induced Smo ciliary translocation

SANT-1 incompletely inhibits cyclopamine binding to cells expressing Smo, and this led to the hypothesis that SANT-1 may change the ability of Smo to interact with cyclopamine [23]. We speculated that this competition between different classes of Smo antagonists could be utilized to control Smo ciliary localization and gain insight into why inactive Smo might traffic to the cilium. In wild-type MEFs, SANT-1 efficiently inhibited cyclopamine- and jervine- induced translocation of Smo to the primary cilium (Fig. 2A). To further investigate competition between SANT-1 and cyclopamine, we utilized *Ptch1* ^−/−^ MEFs, which display constitutive ciliary localization of Smo and Hh pathway activation (Fig. 2E) [15]. Treatment with cyclopamine and jervine inhibited Gli reporter activity in *Ptch1* ^−/−^ MEFs, but did not eliminate Smo staining from the cilium (Fig. 2C, 2D). SANT-1 inhibited the Hh pathway to a similar extent, but drastically reduced the number of Smo^+^ cilia (Fig. 2C, 2D, 2E). Thus, our results suggest that *Veratrum* alkaloids (*e.g.*, cyclopamine) and SANT-1 comprise two distinct classes of Smo inhibitors whose binding to Smo induces conformations that are differentially competent for cilium targeting. Cyclopamine treatment induces an inactive conformation of Smo that is capable of translocating to the primary cilium. Subsequent treatment with SANT-1 could convert inactive Smo into another state that could no longer associate with the cilium. Since SANT-1 can effectively block cyclopamine-induced translocation of Smo to the primary cilium, this suggests that the inactive state of Smo induced by SANT-1 represents the dominant form when both cyclopamine and SANT-1 are present.

The precise mechanism for cyclopamine-induced Smo translocation remains to be elucidated. Cyclopamine-bound Smo may adopt a conformation that allows increased coupling to the anterograde IFT motor subunit Kif3a via β-arrestins [25]. β-arrestins have been shown to mediate the interaction between Smo and Kif3a, thus promoting ciliary localization of Smo and Smo-dependent activation of Gli [25]. The Smo conformation induced by cyclopamine binding may either be incapable of coupling to downstream effectors, or might not interact with retrograde IFT components. Consistent with this latter hypothesis, *Dync2h1* mutant MEFs, which are deficient in retrograde IFT, accumulate Smo on the cilium but do not properly transduce the Hh signal [26].

### Gαs-mediated activation of protein kinase A stimulates translocation of Smo to a proximal region of the primary cilium

Smo structurally resembles a GPCR, but it is currently unclear whether physical coupling of Smo to a Gα subunit is essential for proper Hh signal transduction [27], [28]. Studies in cultured insect and mammalian cells showed that Smo specifically stimulates GTP binding to Gαi family members, and inhibition of Gαi reduces activation of Gli reporters [28]. However, no effect on Hh patterning of the chick neural tube or formation of Gli3 repressor was observed with activation or inhibition of Gαi *in vivo* [27]. Recent data indicates that Gαi impacts Hh transduction in *Drosophila melanogaster* through its effects on PKA activity, although a direct biochemical interaction between Smo and Gαi remains unreported [29]. It is unknown whether GPCR signaling affects Smo localization, either through direct binding to Smo, or through crosstalk via other signaling pathways.

**Figure 3:**
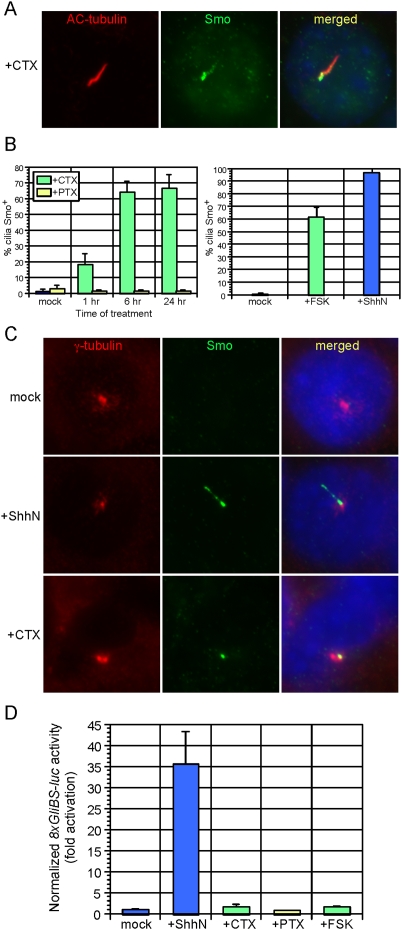
Modulation of Gαs and protein kinase A (PKA) causes Smo accumulation in a proximal region of the primary cilium. (A) Treatment of wild-type MEFs with cholera toxin (CTX) for 24 hours induces Smo (green) translocation to the cilium (red). (B) Quantification of CTX- and FSK-induced Smo ciliary translocation after treatment for the indicated times. Treatment of wild-type MEFs with pertussis toxin (PTX) does not stimulate ciliary translocation of Smo. Error bars indicate +/− SD. (C) Wild-type MEFs were fixed in methanol and stained with antibodies against γ-tubulin (red; label the basal body) and Smo (green). In contrast to the relatively uniform Smo staining on the cilium induced by ShhN, CTX treatment causes Smo accumulation proximal to the basal body. (D) *8xGliBS-luciferase* Hh reporter activity in wild-type MEFs with the indicated treatment. CTX and FSK do not activate the Hh pathway despite inducing ciliary localization of Smo. Data are representative of three independent experiments. Hh reporter activity was normalized to *β-galactosidase* activity produced from a constitutive *hsp68*-lacZ reporter, as alteration of PKA affected *Renilla* luciferase activity (data not shown). Error bars indicate +/− SD.

To investigate whether modulation of Gα subunit activity might impact Smo ciliary trafficking, we treated wild-type MEFs with cholera toxin (CTX), which activates Gαs [30], and pertussis toxin (PTX), which inhibits Gαi [31]. CTX, but not PTX, caused an accumulation of Smo on the primary cilium, which increased with prolonged exposure to the toxin (Fig. 3A, 3B). The CTX-induced Smo distribution was not uniform along the length of the cilium, when compared to ShhN-CM induced Smo translocation (compare Fig. 1A to [Fig pone-0005182-g002] 3A). We fixed MEFs with methanol to better visualize basal bodies, which comprise the base of the primary cilium, and observed that CTX-induced Smo cilium staining was concentrated in a region proximal to the basal body (Fig. 3C). Interestingly, this region appeared similar to a recently defined inversin compartment on the proximal primary cilium [32]. As CTX treatment is expected to activate adenylyl cyclase and thus stimulate PKA, we next asked if direct stimulation of adenylyl cyclase in MEFs with forskolin (FSK) recapitulated CTX-stimulated Smo translocation [33]. FSK treatment resulted in a comparable localization of Smo to the proximal region of the cilium (Fig. 3B, 3C). CTX, PTX, and FSK alone did not activate the Hh pathway significantly as assayed by Gli reporters (Fig. 3D).

**Figure 4:**
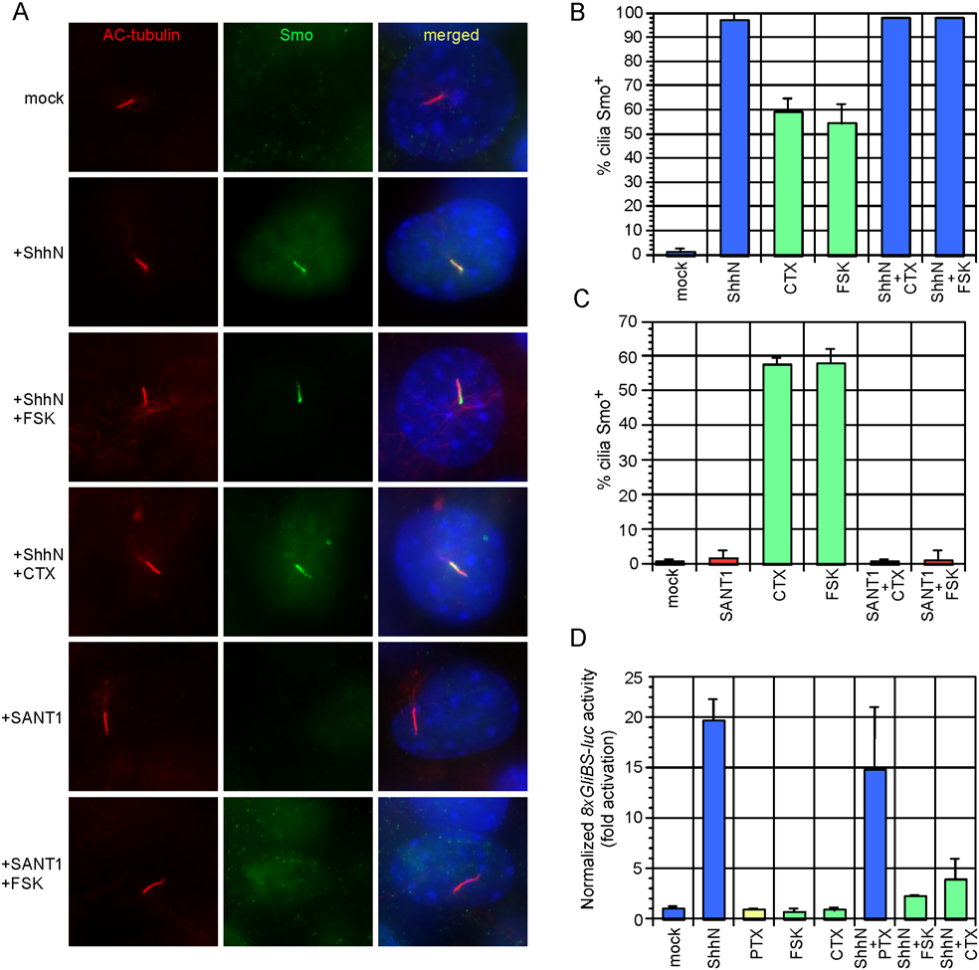
ShhN ligand and SANT-1 act dominantly to PKA-induced Smo translocation. (A) Wild-type MEFs treated with indicated compounds for 24 hours stained with antibodies against acetylated (AC) tubulin (red), Smo (green) and DAPI (blue). ShhN causes Smo distribution along the length of the cilium when PKA is activated, whereas SANT-1 inhibits any type of Smo trafficking on the cilium. (B) Quantification of Smo^+^ cilia in wild-type MEFs after treatment for 24 hours. Nearly all cilia are Smo^+^ when treated with ShhN and CTX or FSK. Error bars indicate +/− SD. (C) Quantification of Smo^+^ cilia in wild-type MEFs after 24 hours. SANT-1 inhibits CTX- and FSK-mediated stimulation of Smo translocation to the proximal region of the primary cilium. Error bars indicate +/− SD. (D) *8xGliBS-luciferase* reporter activity in wild-type MEFs with indicated treatments for 48 hours. CTX and FSK, but not PTX inhibit ShhN stimulation of the reporter. Normalization was performed as in Figure 3. Error bars indicate +/− SD.

We examined the relationship of PKA-mediated Smo translocation to the proximal cilium with ShhN-mediated pathway activation and SANT-1 inhibition. Exposure of MEFs to both ShhN and CTX or FSK resulted in restoration of Smo staining along the entire length of the primary cilium (Fig. 4A, 4B). In contrast, SANT-1 inhibited PKA stimulation of Smo trafficking to the proximal cilium (Fig. 4A, 4C). Two conclusions may be drawn from these results. First, the Smo conformation adopted when bound to SANT-1 is refractory to both cyclopamine- and PKA-stimulated cilium trafficking, suggesting that SANT-1 may act upstream to sequester Smo from or inhibit its interaction with β-arrestins or IFT particles [25]. Second, addition of ShhN overrides accumulation of Smo in the proximal region of the primary cilium. Restricted Smo localization to this region could be a prerequisite for pathway activation, or a means to inhibit Smo trafficking or coupling to downstream components. Consistent with previous reports [34]–[37], we observed that activation of the Hh reporter was blocked by CTX- and FSK-mediated PKA stimulation, but PTX treatment produced only a modest reduction (Fig 4D). We speculate that if PKA positively regulates Smo while promoting Gli repressor production, any potential positive effect on Hh signaling due to partial Smo translocation induced by CTX or FSK may be offset by negative regulation of Gli factors by PKA [38]. Additional studies using endogenous levels of PKA-refractory forms of the Gli proteins will be necessary to rigorously resolve this issue.

*D. melanogaster* Smo utilizes a series of PKA sites in its C-terminal tail to neutralize the inhibitory effects of several clusters of Arg residues [8]. These Arg clusters, but not the PKA sites, are conserved in vertebrates and are critical for maintenance of Smo in an inhibited state that is incapable of trafficking to the cell surface [4], [5]. Further investigation is required to clarify if repression of the autoinhibitory Arg clusters results in Smo ciliary translocation, and if PKA acts directly or indirectly on Smo for targeting to a proximal region of the cilium. Nonetheless, our data raise the possibility that signaling through Gαs-coupled receptors and activation of PKA could influence Smo trafficking both to and on the primary cilium. This could provide a means for crosstalk between signaling pathways.

In this study, we show that Hh pathway agonists, a specific class of antagonists, and PKA activity induce accumulation of Smo in different regions of the primary cilium. Smo localization to the cilium is therefore necessary, but not sufficient, for activation of the Hh pathway. Taken together, the data suggest that Smo can adopt multiple conformations that allow ciliary trafficking, but only a subset of these are competent to activate the Hh cascade. In addition, the precise location of Smo on the primary cilium may be important for activation of the pathway. Further analysis of Smo conformations and identification of factors that communicate these conformations to Gli proteins are necessary for a comprehensive understanding of the role of the primary cilium in Smo function.

## Materials and Methods

### Generation and treatment of cell lines

Mouse embryonic fibroblasts (MEFs) were derived from wild-type embryos at embryonic days (E) 9.5 or E10.5, and from *Ptch1^−/−^* embryos at E9.5. These cells were subsequently immortalized with recombinant retroviruses encoding the simian virus (SV) 40 large T antigen [39]. MEFs were maintained in DMEM (Cellgro) supplemented with 10% fetal bovine serum (Cellgro), L-glutamine (Gibco), penicillin/streptomycin (Gibco), and 200 µg/ml G418 (Gibco). ShhN-conditioned media (CM) was produced as previously described [40]. Briefly, HEK293T cells were transfected with 10 µg of pcDNA3::ShhN using Lipofectamine 2000 (Invitrogen). Conditioned media was harvested 48 and 96 hours post-transfection, pooled, filtered through a 0.22 µm PES syringe filter (Millipore), and buffered with 5 mM HEPES, pH 7.5. Compounds used for treatment of MEFs and their concentrations were as follows: 20-α-hydroxysterol (10 µM, Sigma), 7-β-hydroxysterol (10 µM, Sigma), 7-dehydrocholesterol (10 µM, Sigma), cholecalciferol (pro-vitamin D3, 10 µM, Sigma), cyclopamine (10 µM, Toronto Research Chemicals), jervine (10 µM, Toronto Research Chemicals), SANT-1 (10 µM, Calbiochem), cholera toxin (CTX, 100 ng/µl, Sigma), pertussis toxin (PTX, 100 ng/µl, Calbiochem), forskolin (FSK, 10 µM, Sigma).

### Immunostaining

Confluent MEFs were grown on gelatin-coated glass coverslips (Fisher) and treated with conditioned media or compounds in DMEM supplemented with 0.5% newborn calf serum and penicillin/streptomycin for the indicated times. Cells were washed with PBS, fixed in 4% paraformaldehyde in PBS (Sigma) for 15 min at room temperature, and permeabilized in 0.2% Triton X-100/PBS for 10 min. Alternatively, to better visualize basal bodies, MEFs were fixed in ice-cold methanol for 10 min at −20°C. Blocking was performed in 10% sheep serum/0.02%Triton X-100/PBS for 1 hour at room temperature. Cells were stained with the following primary antibodies in 2% sheep serum/0.02% Triton X-100/PBS at 4°C, overnight: rabbit anti-Smo (1∶500, [17]), mouse anti-acetylated tubulin (1∶2000, Sigma), and mouse anti-γ-tubulin (1∶1000, Sigma). Cells were washed three times in 0.02% Triton X-100/PBS and probed with the following secondary antibodies: mouse Alexa 594 (1∶2000) and rabbit Alexa 488 (1∶2000, Invitrogen). Cells were washed as before, stained with DAPI (1∶10,000, Sigma), and mounted in VectaShield (Vector Laboratories, Burlingame, CA).

### Imaging

Images were taken with a Nikon Eclipse E1000 epifluorescence microscope using a Plan Apochromat 100×/1.40 oil objective (Nikon) and a SPOT 2.3 RT Slider cooled CCD camera. Images were acquired using SPOT Advanced software (Diagnostic Instruments), converted to 24 bit (RGB) images, and the RGB histograms were adjusted to reduce background fluorescence.

### Activity assays

MEFs were seeded in 24-well plates at a concentration of 5×10^4^ cells/ml the day prior to transfection. Fugene 6 (Roche) was used for transfection of reporter constructs according to manufacturer's instructions. We transfected a mix of pcDNA3 (Invitrogen) : 8xGliBS-firefly luciferase [24] : pRL-TK (Promega) or *hsp68*-lacZ with a ratio of 4∶5∶1. 48 hours post-transfection, media was changed to low serum, and ShhN-CM or compounds were added for 36–48 hours. Cells were harvested and reporter activity measured using a Dual Luciferase kit (Promega) and a Luminescent β-galactosidase detection kit II (Clontech), on an LmaxII 384 luminometer (Molecular Devices).
